# Automatic segmentation of multiple cardiovascular structures from cardiac computed tomography angiography images using deep learning

**DOI:** 10.1371/journal.pone.0232573

**Published:** 2020-05-06

**Authors:** Lohendran Baskaran, Subhi J. Al’Aref, Gabriel Maliakal, Benjamin C. Lee, Zhuoran Xu, Jeong W. Choi, Sang-Eun Lee, Ji Min Sung, Fay Y. Lin, Simon Dunham, Bobak Mosadegh, Yong-Jin Kim, Ilan Gottlieb, Byoung Kwon Lee, Eun Ju Chun, Filippo Cademartiri, Erica Maffei, Hugo Marques, Sanghoon Shin, Jung Hyun Choi, Kavitha Chinnaiyan, Martin Hadamitzky, Edoardo Conte, Daniele Andreini, Gianluca Pontone, Matthew J. Budoff, Jonathon A. Leipsic, Gilbert L. Raff, Renu Virmani, Habib Samady, Peter H. Stone, Daniel S. Berman, Jagat Narula, Jeroen J. Bax, Hyuk-Jae Chang, James K. Min, Leslee J. Shaw

**Affiliations:** 1 Dalio Institute of Cardiovascular Imaging, Weill Cornell Medicine, New York, New York, United States of America; 2 Department of Radiology, New York-Presbyterian Hospital and Weill Cornell Medicine, New York, New York, United States of America; 3 Department of Cardiovascular Medicine, National Heart Centre, Singapore, Singapore; 4 Cleerly, Inc, New York, New York, United States of America; 5 Division of Cardiology, Severance Cardiovascular Hospital, Integrative Cardiovascular Imaging Center, Yonsei University College of Medicine, Seoul, South Korea; 6 Division of Cardiology, Department of Internal Medicine, Ewha Womans University Seoul Hospital, Seoul, Korea; 7 Department of Internal Medicine, Seoul National University College of Medicine, Cardiovascular Center, Seoul National University Hospital, Seoul, South Korea; 8 Department of Radiology, Casa de Saude São Jose, Rio de Janeiro, Brazil; 9 Division of Cardiology, Gangnam Severance Hospital, Yonsei University College of Medicine, Seoul, Korea; 10 Department of Radiology, Seoul National University Bundang Hospital, Sungnam, South Korea; 11 Cardiovascular Imaging Center, SDN IRCCS, Naples, Italy; 12 Department of Radiology, Area Vasta 1/ASUR Marche, Urbino, Italy; 13 UNICA, Unit of Cardiovascular Imaging, Hospital da Luz, Lisboa, Portugal; 14 Pusan University Hospital, Busan, South Korea; 15 Department of Cardiology, William Beaumont Hospital, Royal Oak, Michigan, United States of America; 16 Department of Radiology and Nuclear Medicine, German Heart Center Munich, Munich, Germany; 17 Centro Cardiologico Monzino, IRCCS, Milan, Italy; 18 Department of Medicine, Los Angeles Biomedical Research Institute, Torrance, California, United States of America; 19 Department of Medicine and Radiology, University of British Columbia, Vancouver, British Columbia, Canada; 20 Department of Cardiology, William Beaumont Hospital, Royal Oak, Michigan, United States of America; 21 Department of Pathology, CVPath Institute, Gaithersburg, Maryland, United States of America; 22 Division of Cardiology, Emory University School of Medicine, Atlanta, Georgia, United States of America; 23 Cardiovascular Division, Brigham and Women’s Hospital, Harvard Medical School, Boston, Massachusetts, United States of America; 24 Department of Imaging and Medicine, Cedars Sinai Medical Center, Los Angeles, California, United States of America; 25 Icahn School of Medicine at Mount Sinai, Mount Sinai Heart, Zena and Michael A. Wiener Cardiovascular Institute, and Marie-Josée and Henry R. Kravis Center for Cardiovascular Health, New York, New York, United States of America; 26 Department of Cardiology, Leiden University Medical Center, Leiden, the Netherlands; Medizinische Universitat Graz, AUSTRIA

## Abstract

**Objectives:**

To develop, demonstrate and evaluate an automated deep learning method for multiple cardiovascular structure segmentation.

**Background:**

Segmentation of cardiovascular images is resource-intensive. We design an automated deep learning method for the segmentation of multiple structures from Coronary Computed Tomography Angiography (CCTA) images.

**Methods:**

Images from a multicenter registry of patients that underwent clinically-indicated CCTA were used. The proximal ascending and descending aorta (PAA, DA), superior and inferior vena cavae (SVC, IVC), pulmonary artery (PA), coronary sinus (CS), right ventricular wall (RVW) and left atrial wall (LAW) were annotated as ground truth. The U-net-derived deep learning model was trained, validated and tested in a 70:20:10 split.

**Results:**

The dataset comprised 206 patients, with 5.130 billion pixels. Mean age was 59.9 ± 9.4 yrs., and was 42.7% female. An overall median Dice score of 0.820 (0.782, 0.843) was achieved. Median Dice scores for PAA, DA, SVC, IVC, PA, CS, RVW and LAW were 0.969 (0.979, 0.988), 0.953 (0.955, 0.983), 0.937 (0.934, 0.965), 0.903 (0.897, 0.948), 0.775 (0.724, 0.925), 0.720 (0.642, 0.809), 0.685 (0.631, 0.761) and 0.625 (0.596, 0.749) respectively. Apart from the CS, there were no significant differences in performance between sexes or age groups.

**Conclusions:**

An automated deep learning model demonstrated segmentation of multiple cardiovascular structures from CCTA images with reasonable overall accuracy when evaluated on a pixel level.

## Introduction

In evaluating cardiovascular disease (CVD), the imaging of structures plays a key role in diagnosis, as well as in surveillance of progression. Coronary Computed Tomography Angiography (CCTA) provides isotropic high spatial resolution imaging non-invasively. In both research and clinical workflows, the necessary quantitative and qualitative evaluation of these structures is assisted via available commercial software packages. However, this requires manual input, rendering this process time-consuming and operator-dependent [1].

As it uses large amounts of information to build predictive models via novel algorithmic strategies, machine learning (ML) is well-suited to imaging, and its role in the cardiovascular space is expanding [2]. Deep learning is a subdomain of ML that uses sophisticated frameworks comprising networks with many intermediate layers of “neurons” to perform automated feature extraction. This results in the ability to map inputs to outputs via complex pathways [3]. Here, we apply deep learning to CCTA images to develop, demonstrate and evaluate an automated model for the identification of multiple cardiovascular structures.

## Methods

### Study population

The population consisted of a convenience sample randomly selected from an international, multicenter, prospective, observational registry that has been described previously [4,5]. Inclusion criteria were patients undergoing clinically indicated CCTA, with images of sufficient quality to be annotated. Known coronary artery disease (CAD), hemodynamic instability, arrhythmia, and uninterpretable CCTA were exclusion criteria. Each site obtained local institutional review or ethics board approval.

### Image acquisition and segmentation

Scanners were ≥ 64-detector rows, and acquisition, post-processing and interpretation were performed to current guidelines [6].

Images were obtained and reconstructed at 0.50 mm thickness. Files in Digital Imaging and Communications in Medicine (DICOM) format were transmitted to a core laboratory, and structure annotation was done in a blinded manner by level III–experienced technologists. The luminal segments of eight cardiovascular structures were annotated from the iodinated contrast-tissue border interface using Adobe Photoshop (Adobe Systems, San Jose, California): the proximal ascending and descending aorta (PAA, DA), superior and inferior vena cavae (SVC, IVC), pulmonary artery (PA), coronary sinus (CS), right ventricular wall (RVW) and left atrial wall (LAW). The superior and inferior axial limits of all structures were limited to within the guideline-recommended scan range, from below the tracheal bifurcation or the mid-level of the left pulmonary artery and extending below the cardiac border [6], and any remaining segments of structures outside this range were not analyzed. The PAA was identified as originating at the plane corresponding to the nadirs of all 3 aortic valve cusps, to the plane most proximal to the origin of the brachiocephalic artery. The DA was defined as originating immediately distal to the origin of the left subclavian artery, extending to the most inferior axial slice. The vena cavae were identified as being venous vessels coursing along the right middle mediastinum, adjacent to and to the right of the trachea and PAA and draining into the right atrium [7,8]. The PA included the main, left and right pulmonary arteries. The CS was identified as the cardiac venous structure continuing in the atrioventricular groove from the great cardiac vein, adjacent to left circumflex coronary artery and draining into the right atrium [9]. RVW was defined as the right ventricular myocardial volume derived by the delineation of its endocardial and epicardial borders, excluding papillary muscles and trabeculations, and followed the contours below the atrioventricular valve planes on a three-dimensional isotropic voxel level. LAW identification used the left atrial myocardial volume derived by the delineation of its endocardial and epicardial borders and included the appendage but excluded adjacent veins. These annotations, established and verified by board certified cardiologists, were used as the “ground truth” for the deep learning model.

### Splitting of dataset and preprocessing

The entire dataset was split into three parts; training (70%), validation (20%) and testing (10%). No two parts contained images from the same patient. An open source python library known as ‘psd_tools’ was used for the extraction of the ground truth from the annotated images [10]. Annotated slices were extracted from the ground truth Photoshop files and arranged according to each label/structure’s assigned color code. DICOM volumes were converted to have an isotropic voxel spacing of 0.625 mm x 0.625 mm x 0.625 mm, with the same volumetric resolution for the extracted labels. The images were windowed with a Hounsfield unit (HU) window (-300,500) so that all structures of interest were optimally visible. Each input image contained a pair of background and foreground label images. All the images and corresponding labels were then resized to 512x512 pixels and passed to the model.

### Deep learning model

**Fig 1:**
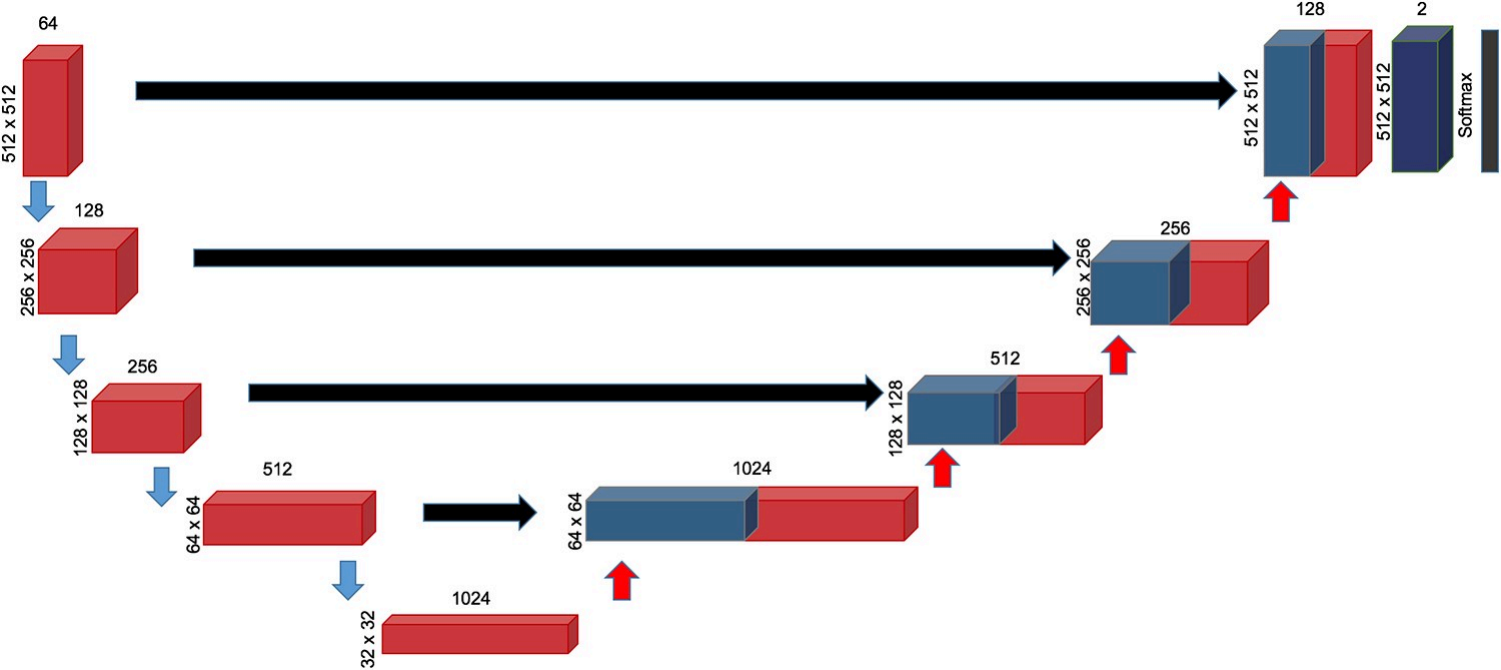
The deep learning network architecture. Each red block represents two consecutive sets of 3x3 Convolution layer, ReLU activation and Batch Normalization. The number of feature maps in each layer is on top of each box, while the size of each feature map per layer is indicated on the left. Finally, the feature maps are passed through a softmax layer to obtain pixel wise probabilities of belonging to the given class to obtain the outputs.

As it has previously been used for medical image segmentation in thoracic images, a convolutional neural network (CNN), U-Net was used for the deep learning architecture [11,12]. U-net comprises 4 layers (Fig 1). The image is first down-sampled by a Conv3x3 layer consisting of two runs through a set comprising of a convolution with 3x3 kernel, Rectified Linear Unit (ReLU) and a batch normalization layer. The output feature maps from this layer are further down sampled by half the resolution. After 4 layers of this, the feature maps are now up sampled by transposed convolution (kernel size of 2 and a stride of 2 followed by successive Conv3x3 blocks). The feature maps from the contracting path are concatenated with those of the expanding path. At the final layer the feature maps are reduced from 128 to 2 using a Conv 1x1 block which consists of a 1x1 convolutional kernel, and pixel-wise probabilities for belonging to each class is obtained once this is passed to a Softmax layer. Eight similar but separate networks were trained for each structure. Prior work on other cardiovascular structures using this framework has previously been reported [13].

### Training strategy and model evaluation

**Fig 2:**
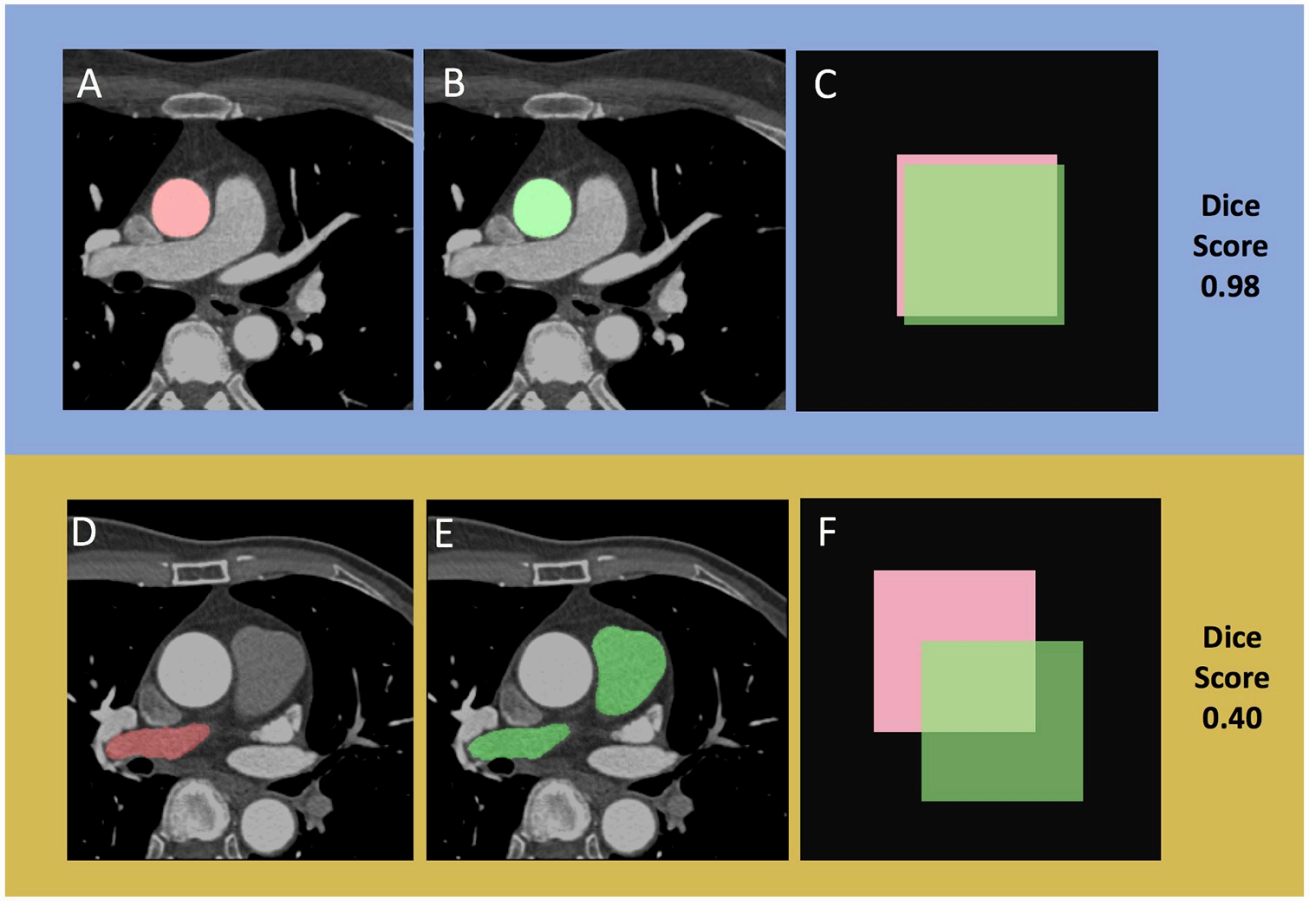
Dice score visualization. The Dice score is used to gauge model performance, ranging from 0 to 1. 1 corresponds to a pixel perfect match between the deep learning model output (red, A and D) and ground truth annotation (green, B and D). The model output with the higher Dice score (A) had greater overlap (C) with the ground truth (B) than the output (D) that had lesser overlap (F), as it did not predict that main pulmonary artery that was annotated in (E).

The images were randomly shuffled and passed to the network (batch size 4, resolution 512x512). The network output was binary masks for two classes i.e. the foreground and the background. The Dice loss, obtained by subtracting the mean Dice similarity score from 1, was used to train the network [14]. Training used Adam optimizer with a learning rate of 0.001 to carry out training [15]. Outputs were compared with the ground truth which contains complimentary images of the contour of interest using the Dice loss. The network having the lowest Dice loss on the validation set amongst the epochs (one cycle through the full training dataset) was selected and evaluated on the test set. Data was shuffled every epoch. Each model was trained for 50 epochs, and the model with the best validation loss was chosen among these epochs. Image-based performance metric was based on Dice loss, calculated by subtracting the mean Dice similarity score from 1 [14]. This score quantifies the pixel-wise degree of similarity between the model predicted segmentation mask and the ground truth, and ranges from 0 (no similarity) to 1 (identical) (Fig 2), mathematically expressed as:

$$Dice\;similarity\;coefficient=\frac{\left(2*True\;Positive\right)}{\left(2*True\;Positive+False\;Positive+False\;Negative\right)}$$

### Statistical analysis

Statistical analysis was performed using Python 3.7 using the scikit-learn library. Continuous and normally distributed variables were expressed by mean ± standard deviation. Categorical data were expressed by number and percentage. Dice scores were summarized as medians and quartiles. Subgroup analysis of Dice scores by gender and age were compared by Wilcoxon test. A P value of <0.05 was considered significant.

## Results

The study comprised 206 patients, with 19,572 images and 5.130 billion pixels. Cohort mean age was 59.9 ± 9.4 yrs., and was 42.7% female (Table 1). Prevalence of diabetes, dyslipidemia, hypertension and smoking were 22.22%, 34.78%, 44.93% and 64.73% respectively. The training set comprised 144 patients (13701 images), validation set 42 patients (3914 images) and testing set 20 patients (1957 images). There were no differences for patient characteristics between the training, validation and test sets (Table 2).

**Table 1:** Baseline characteristics.

Characteristic	Total
**N**	**206**
Age, years (SD)	59.85, 9.41
Female (%)	42.72
Diabetes Mellitus (%)	22.22
Dyslipidemia (%)	34.78
Hypertension (%)	44.93
Smoker (%)	64.73

**Table 2:** Baseline characteristics by dataset.

Characteristic	Training	Validation	Testing	*P* (training vs validation)	*P* (validation vs testing)	*P* (training vs testing)
**N**	**144**	**42**	**20**			
Age, years (SD)	59.95 (9.66)	60.43 (7.78)	57.95 (10.38)	0.744	0.362	0.434
Female (%)	45.14	33.33	45	0.167	0.396	0.991
Diabetes (%)	23.61	16.67	25	0.311	0.474	0.896
Dyslipidemia (%)	34.72	42.86	20	0.353	0.063	0.153
Hypertension (%)	45.14	45.24	45	0.99	0.986	0.991
Smoker (%)	62.86	76.19	70	0.092	0.622	0.532

**Fig 3:**
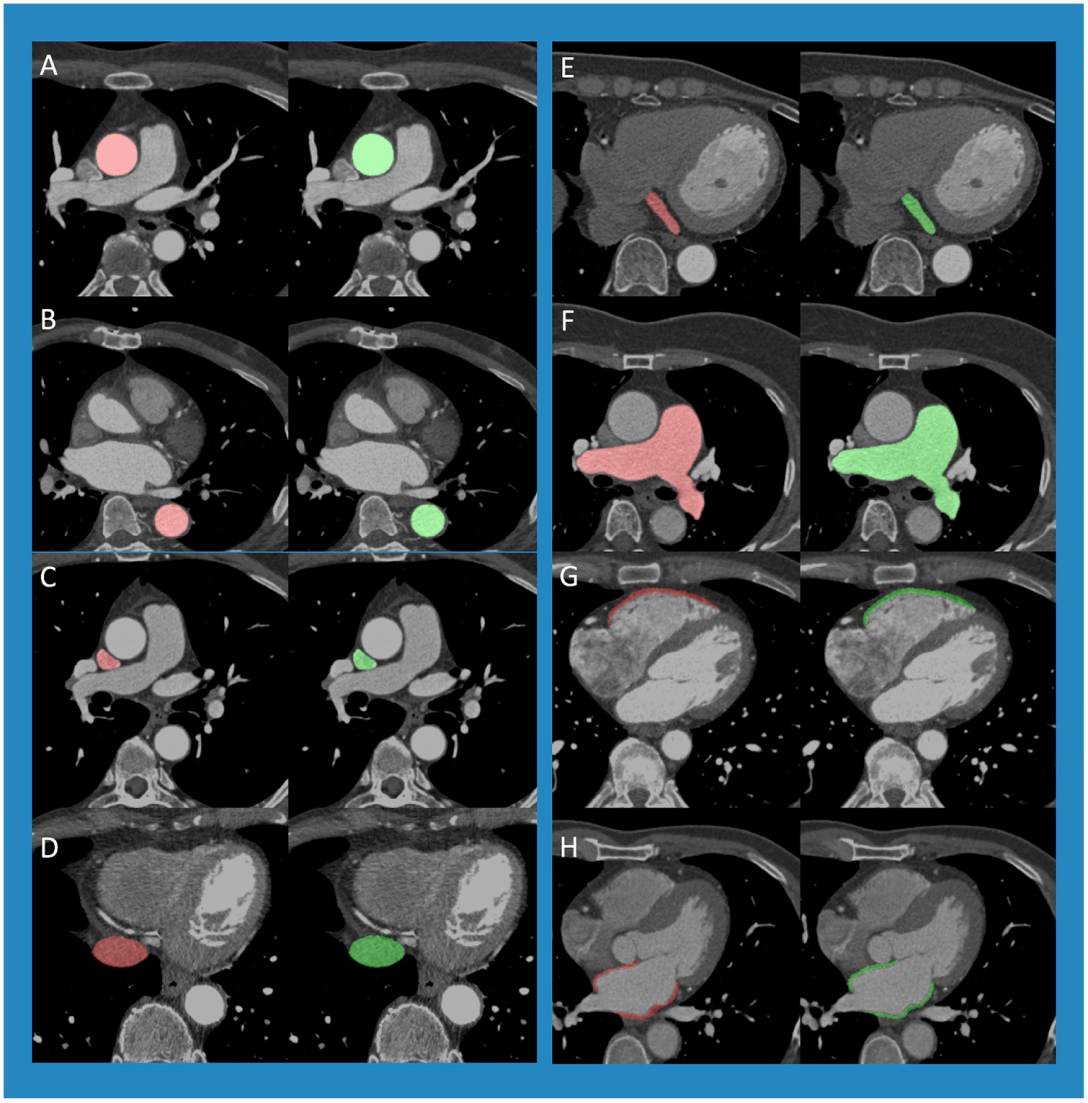
Demonstrative comparisons between the ground truth manual annotation (green) and prediction overlaid on the image(red) are shown for the: A) PAA, B) DA, C) SVC, D) IVC, E) CS, F) PA, G) RVW and H) LAW.

A combined overall median Dice score of 0.820 (interquartile range: 0.782, 0.843) was achieved on the validation set for the structures (PAA, DA, SVC, IVC, PA, CS, RVW, LAW) and demonstrative comparisons between the original image, manual annotation and model prediction are shown in Fig 3. Median Dice scores for PAA, DA, SVC, IVC, PA, CS, RVW and LAW were 0.969 (interquartile range: 0.979, 0.988), 0.953 (interquartile range: 0.955, 0.983), 0.937 (interquartile range: 0.934, 0.965), 0.903 (interquartile range: 0.897, 0.948), 0.775 (interquartile range: 0.724, 0.925), 0.720 (interquartile range: 0.642, 0.809), 0.685 (interquartile range: 0.631, 0.761) and 0.625 (interquartile range: 0.596, 0.749) respectively (Table 3). For PAA, there were no significant differences between sexes (male = 0.954, female = 0.987) or age groups (age<65 = 0.969, age ≥65 = 0.968). For the DA, there were no significant differences in Dice scores between sex- or age-based subgroups (male = 0.949, female = 0.956, age<65 = 0.923, age ≥65 = 0.965), nor were there for the SVC (male = 0.929, female = 0.945, age<65 = 0.946, age ≥65 = 0.929), IVC (male = 0.891, female = 0.918, age<65 = 0.912, age ≥65 = 0.896), PA (male = 0.773, female = 0.778, age<65 = 0.738, age ≥65 = 0.799), RVW (male = 0.669, female = 0.702, age<65 = 0.699, age ≥65 = 0.676), or the LAW (male = 0.586, female = 0.668, age<65 = 0.630, age ≥65 = 0.620) (*P* > 0.05 for all). For the CS, although there was no significant difference in model prediction performance between scores for age<65 (0.756) and age ≥65 (0.709) (*P* = 0.365), there was a difference between the scores for male (0.784) and female (0.670) patients (*P* = 0.048). Automated segmentation for all 8 structures took 19.37 seconds per patient, at 0.198 seconds per slice, whereas manual segmentation took approximately 1 hour per patient.

**Table 3:** Model performance.

Structure	Category	Value		*P*
		Median	Quartiles (25^th^, 75th)	
**PAA**	Overall	0.969	(0.979, 0.988)	-
	Male	0.954	(0.947, 0.985)	0.086
	Female	0.987	(0.986, 0.990)	
	Age < 65 years	0.969	(0.966, 0.986)	0.987
	Age ≥ 65 years	0.968	(0.984, 0.988)	
**DA**	Overall	0.953	(0.955, 0.983)	-
	Male	0.949	(0.924, 0.977)	0.828
	Female	0.956	(0.974, 0.985)	
	Age < 65 years	0.923	(0.937, 0.978)	0.340
	Age ≥ 65 years	0.965	(0.971, 0.983)	
**SVC**	Overall	0.937	(0.934, 0.965)	-
	Male	0.929	(0.939, 0.965)	0.494
	Female	0.945	(0.934, 0.966)	
	Age < 65 years	0.946	(0.930, 0.967)	0.494
	Age ≥ 65 years	0.929	(0.960, 0.965)	
**IVC**	Overall	0.903	(0.897, 0.948)	-
	Male	0.891	(0.893, 0.951)	0.495
	Female	0.918	(0.02, 0.939)	
	Age < 65 years	0.912	(0.901, 0.937)	0.660
	Age ≥ 65 years	0.896	(0.901, 0.951)	
**PA**	Overall	0.775	(0.724, 0.925)	-
	Male	0.773	(0.719, 0.907)	0.968
	Female	0.778	(0.758, 0.928)	
	Age < 65 years	0.738	(0.716, 0.899)	0.569
	Age ≥ 65 years	0.799	(0.746, 0.932)	
**CS**	Overall	0.720	(0.642, 0.809)	-
	Male	0.784	(0.754, 0.857)	0.048
	Female	0.670	(0.586, 0.774)	
	Age < 65 years	0.756	(0.744, 0.771)	0.365
	Age ≥ 65 years	0.709	(0.563, 0.821)	
**RVW**	Overall	0.685	(0.631, 0.761)	-
	Male	0.669	(0.636, 0.758)	0.519
	Female	0.702	(0.651, 0.754)	
	Age < 65 years	0.699	(0.665, 0.734)	0.622
	Age ≥ 65 years	0.676	(0.595, 0.761)	
**LAW**	Overall	0.625	(0.596, 0.749)	-
	Male	0.586	(0.596, 0.749)	0.188
	Female	0.668	(0.636, 0.763)	
	Age < 65 years	0.630	(0.567, 0.745)	0.830
	Age ≥ 65 years	0.620	(0.634, 0.749)	

Abbreviations: PAA = proximal ascending aorta, DA = descending aorta, SVC = superior vena cava, IVC = inferior vena cava, PA = pulmonary artery, CS = coronary sinus, RVW = right ventricular wall, LAW = left atrial wall

## Discussion

This study demonstrated the capability of a deep learning model to rapidly identify the majority of the great vessels, the coronary sinus, and the left atrial and right ventricular walls in an automated, pixel-wise manner. This was done within this multicenter, international cohort with reasonable accuracy overall, agreeing well with manual annotation across sex- and age -stratified subgroups.

This model was able to identify the thoracic aorta (PAA and DA) with high accuracy on a pixel level, as reflected by high Dice scores. A prior study using deep learning on 331 abdominal CTs segmented the abdominal aorta with a mean Dice score of 0.796 [16]. However, this performance was on the validation set, rather than on an unseen testing set, as compared to the current study. Another study using dilated CNNs segmented three parts of the thoracic aorta (PAA, aortic arch and DA) and obtained Dice scores of 0.83–0.88 [17]. Although the dataset used only 24 scans with two-fold cross validation, the Dice score was commendable as the CT scans were non-contrast and non-gated. Our study obtained Dice scores of 0.969 and 0.953 for the PAA and DA respectively, and that may be partly attributable to the use of contrast and ECG gating that may have better delineated the border between the vessel wall and lumen. This enhanced edge and visual boundary detection may assist in CNN-based segmentation tasks [18]. Other studies on chest CTs obtained Dice scores of 0.93–0.95 [19–21]. However, these were label- or atlas-based rather than deep learning-based, and segmented the thoracic aorta as a whole, rather than distinguishing the PAA from the DA. In a large open-access challenge, Dice scores for the PAA, obtained using deep learning-based techniques, were similar to ours [22]. As the PAA and DA are large tubular structures are already easily identified by the radiologist, the potential added value of an expansion of the current method would be to detect abnormalities along these structures in a rapid manner as well as to quantify measurable parameters, e.g. to identify a larger cross-sectional diameter indicative of an aortic aneurysm. To our knowledge, this is the first demonstration of deep learning-based segmentation on both the PAA and DA in cardiac gated contrast-enhanced scans.

**Fig 4:**
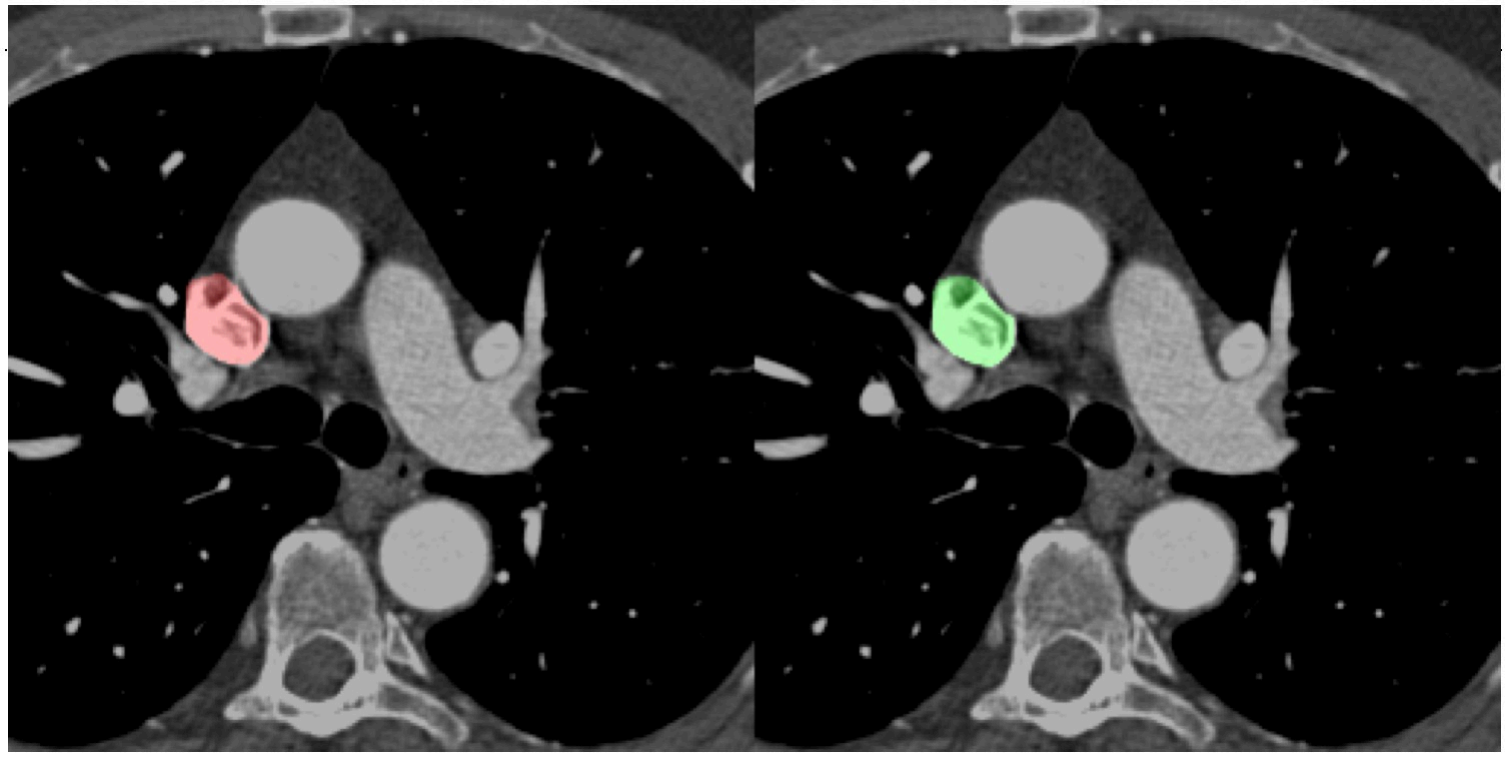
Accurate prediction of the SVC despite suboptimal contrast opacification. The ground truth annotation is in green and the model prediction in red. Abbreviations: SVC = superior vena cava.

Although the IVC has previously been segmented, it was via the use of masks that required manual input [23]. That study obtained a Dice score of 0.896. Conversely, the current study is automated, and obtained a higher score of 0.903 for the IVC and 0.937 for the SVC, congruent with the overall performance advantage of deep learning methods over atlas-based algorithms in the segmentation of other cardiovascular structures [22]. This performance is despite vena cavae visualization that is below ideal (Fig 4), due to the irregular shape and suboptimal contrast timing, as the contrast for the scans in this study were optimized for coronary artery opacification, rather than the vena cavae. Although the SVC and IVC are easily identified by the radiologist, the current method potentiates measurements of the vessels required for insertion of stents, or for the rapid identification of pathology, e.g. thrombi. To the best of our knowledge, this is the first attempt to use deep learning to segment the vena cavae.

The indications for imaging and segmentation of the CS have broadened in parallel with developments in left ventricular pacing, arrhythmia ablation and transcatheter mitral valve replacements and repairs. The difficulty in identifying the irregularly-shaped CS, especially with suboptimal contrast opacification, may account for the lower Dice score of 0.720 when compared to the other vessels. When compared to other tubular cardiovascular structures, this disparity in segmentation performance is congruent with a prior study. In a study using model-based frameworks to segment multiple cardiovascular structures from 35 CT scans, segmentation obtained a volume overlap of 0.952 on the aorta, but only 0.704 on the CS [24]. Furthermore, in our study, there was an additional deterioration in performance in images of female subject compared to male. Whilst this may partly due to the generally smaller size of CS in females, further external validation is required to address this. To the best of our knowledge, the current study is the first to use CNN-based deep learning to segment the CS, providing proof of feasibility. This performance is expected to improve with datasets, using specialized CS imaging protocols.

Imaging of the PA may aid in the identification and prognostication of pulmonary hypertension. In the study using model-based frameworks, segmentation of the PA obtained a volume overlap of 0.940 [24]. Although not using deep learning, that performance is superior than the current study’s performance, with a Dice score of 0.775. In another study comprising an open challenge (using both atlas- and deep learning-based models), segmentation of the PA obtained a Dice score of 0.80 [22]. These results suggest that a deep learning approach may not be the best solution to segmentation of the PA, whose variability in terms of shape and appearance is notably greater than structures such as the DA. Additionally, our current model employs a 2D CNN model, that does not incorporate information in serial images along the z-axis. The models previously mentioned incorporate 3D information, that may aid in segmentation. This limitation of our current study is scope for further development.

When compared to the tubular structures, both the RVW and LAW have poorer performance. This may be due to the irregular shape and thin walls for these structures. Although prior studies have segmented the right ventricle, these studies have not distinguished between the blood pool or volume and the wall [22,24]. In contradistinction, to the best of our knowledge, this is the first demonstration of a deep learning-based model to segment the RVW separately. To our knowledge, this is the first demonstration of a deep learning-based LAW segmentation model.

**Fig 5:**
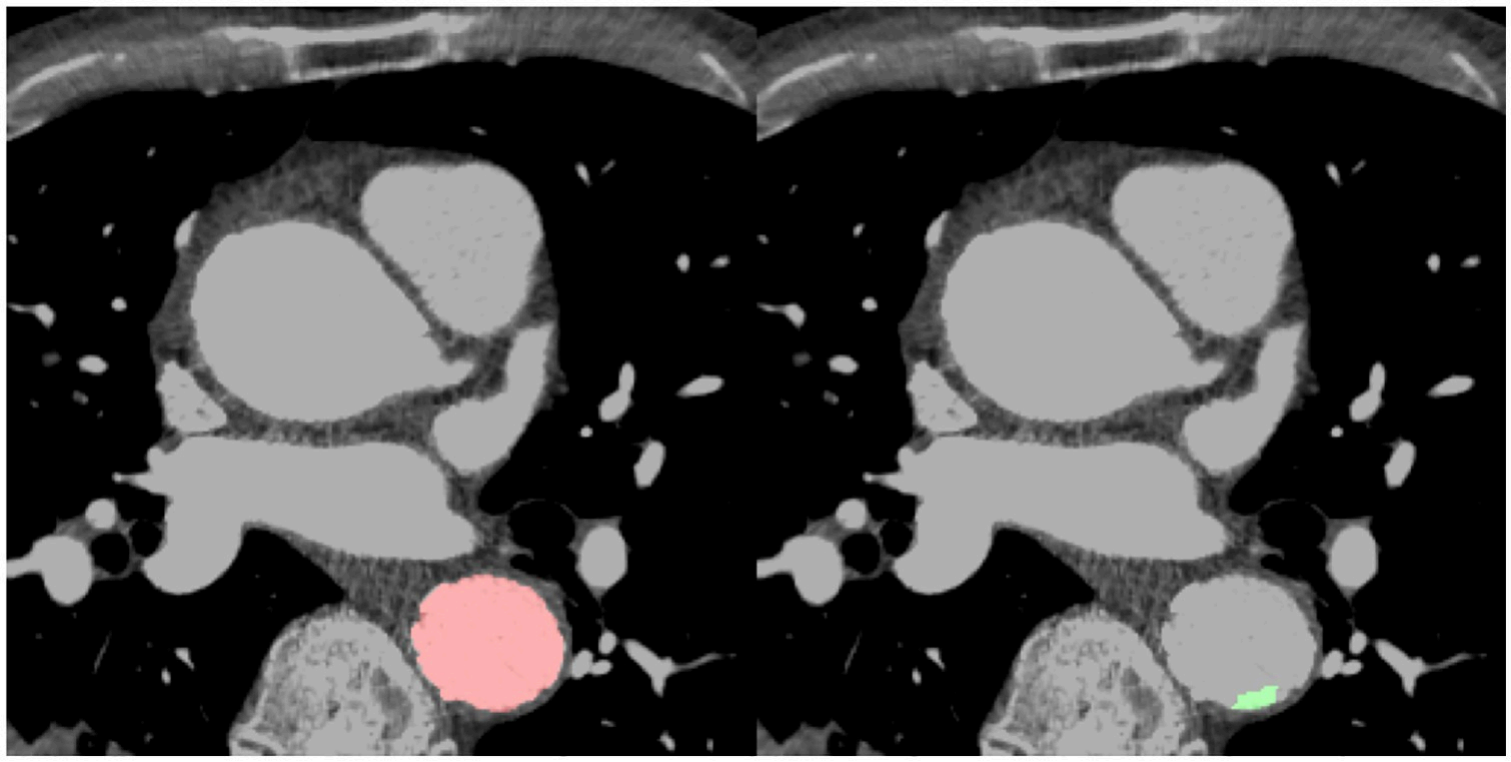
An example of the model as a “second reader”, correcting human error. The DA was partially missed during that were missed during manual annotation (green). The model correctly identified these (red), but obtained a lower Dice score as it did not match the ground truth set during manual annotation. Abbreviations: DA = descending aorta.

A deep learning model for multiple structures would be consistent, reproducing the same result every time. During this study, certain model outputs obtained lower Dice scores, but on further inspection, this was due to ground truth annotation error by human readers, rather than by the model (Fig 5). This study introduces the feasibility of a deep-learning model as a “second reader”. A second reader reduces interpretative error, and results in changes in decision with meaningful clinical impact. Deep learning could contribute with minimal time and cost issues, improving its feasibility. The rapid throughput could allow future integration into clinical workflows with minimal disruption, helping alleviate the clinical diagnostic burden [25] As such, this study tentatively raises the possibility of clinical integration to enhance diagnostic speed, lower costs and reduce error.

There are limitations to the current study. Whilst predicting 8 structures, this model omitted the four cardiac chambers and the left ventricular wall. However, these structures have previously been segmented by our group, and this current study is an extension of that work [13]. Although segmenting most of the great vessels, the current study does not segment the pulmonary veins. The pulmonary veins are of therapeutic interest, as potential sites for arrhythmia ablation. The anatomy of the pulmonary veins is variable, as are its boundaries, and mapping of these structures for ablation planning could be possible, with modification of the current model. Further work could include prediction of the pulmonary veins, which has been segmented previously [24]. The scan timing of these images did not optimize contrast opacification for a number of structures, including the vena cavae, RVW and CS. The current study did not include 3D information in the CNN, and future developments to incorporate this will likely improve performance. An additional limitation is the study size. There was a “hold-out” 10% test set that the model never “saw” until final performance evaluation. Whist this comprised only 20 patients, this set comprised 1957 images, a number deemed more than adequate for medical image-based deep learning applications [26]. The high Dice score attests to the model’s robustness between the training, validation and testing cohorts, but cannot exclude overfitting. Overfitting, whilst not excluded, may be less likely as the model did make incorrect predictions (Fig 2). A larger study that serves as external validation will address these limitations and may improve performance.

Concluding, an automated deep learning model demonstrated segmentation of cardiovascular structures from CCTA images with reasonable overall accuracy when evaluated on a pixel level, and to the best of our knowledge, is the first demonstration of deep learning to segment the SVC, IVC, CS, RVW and LAW. This heralds its integration into research and clinical workflows.

## Abbreviations

CCTA: Coronary Computed Tomography Angiography
CNN: Convolutional Neural Network
CS: Coronary sinus
CVD: Cardiovascular disease
DA: Descending aorta
DICOM: Digital Imaging and Communications in Medicine
HU: Hounsfield unit
IVC: Inferior vena cava
LAW: Left atrial wall
ML: Machine learning
PA: Pulmonary artery
PAA: Proximal ascending aorta
RVW: Right ventricular wall
SVC: Superior vena cava
